# The Conventions at Buffalo

**Published:** 1905-09

**Authors:** 


### Editorial.

### THE CONVENTIONS AT BUFFALO.

The conventions held at this season of the year are, in one sense, a professional thermometer, indicating, through the rise and fall of the spirit manifested, the temperature of the national body. It may not always be truly indicative of the character of the impulses governing the entire number of the 25,000 practitioners in this country, but, while possibly defective in this respect, it is the only standard of judgment we can use as the years go by.

The present period seems to mark an advance as far as the National Dental Association is concerned, for, notwithstanding there were many criticisms regarding its value, these, it seemed to the writer, were not altogether just. The papers presented were, upon the whole, of better quality than we are generally called upon to hear on similar occasions. Yet, while this is true, there should be a greater cultivation of the critical spirit in those having charge of this matter and eliminate all essays that have not, at least, a flavor of originality.

It was given out that the experiment of holding but one section daily would be tried. Why this was not done is not known, but it was unfortunate that the opportunity was not grasped. The experience of running several sections at the same time had been tried previously at the Columbian Congress and at St. Louis, and had in each instance proved a dismal failure. The holding of one section daily promised good results, as it would have brought all together at the same time and place, and virtually would have become a general meeting. The officers controlling this matter should seriously consider this problem, and either abandon the sections absolutely or else arrange these, as stated, and confine the essays to the number necessary to fulfil the daily needs of the association.

The number in attendance was larger than expected. The counter attraction on the Pacific Coast, and the State conventions at this season, necessarily kept many away, yet the interest was sustained from the first day to the last. The climatic conditions were all that could be desired. The cool weather was particularly grateful to the parboiled denizens of the Atlantic Coast, and this contributed not a little to the energy and life of the meeting.

It was unfortunate, in some respects, that the clinics were forced so far away from the general meeting-place. The Buffalo Dental College was, however, well adapted for the purpose, and the crowds in attendance attested to the interest. This contributed, in a large degree, to reduce attendance at the sections and general meetings. Unstinted praise must be given to Dr. Wedelstaedt for his untiring labor in producing one of the best managed clinical exhibits it has been the pleasure of the writer to see. From the lower floor to the upper the rooms were filled with workers. It is rare to find so many fine gold operators brought together as upon this occasion. This was especially satisfactory in view of the fact that the porcelain craze has, for some time past, thrown the old reliable methods somewhat in the shadow. We cannot afford to lose any of the good things that have been proved of value in the past.

Valuable as these clinics are from a practical view, they must become a serious problem for the future in connection with dental conventions. With the divided interest between clinics, trade exhibits, and sectional meetings, something must suffer loss. The interest, with the average operator, lies naturally with the practical, and the meetings are more and more taking a second place, and the scientific side of dentistry is a sufferer thereby. The solution of this serious matter is not, at present, attainable. The Section on Stomatology, American Medical Association, has settled this question for itself, but in eliminating all practical matters it has developed a one-sided dentistry, excellent in itself, but impossible as a means of perfecting dental practitioners.

The National will meet next year at Atlanta, Ga. It was necessary under the rules that it should go south next year, and this city seemed the most available place. Meeting in the early September is, in the view of the writer, a grave error. Very few members from the North will be able to attend at that period. The vacation time is practically over, and few will care to extend it beyond September 1.

The most important matter decided upon by this national body was the decision to publish a journal as the organ of the association. This matter has been under consideration for the past two years, it having been placed in the charge of a committee at the meeting held at Asheville, N. C., in 1903. This committee was unable to report in 1904, as the National Association passed its regular meeting in deference to the Fourth International Dental Congress.

The report was very fully considered in council, and was finally sent to the general meeting, and it was there unanimously decided in favor of establishing a journal independent in character and representing the interests of the dental profession in this country. A committee of five was appointed to arrange for the early publication of this journal upon a permanent financial basis. It is probable that this committee will mature its plans during this early fall, so that the journal can be launched on its career of hoped-for usefulness at the beginning of the year.

The National Association of Dental Examiners held its annual meeting at Buffalo, but, as these meetings are held with closed doors, it is always difficult to learn the result of its deliberations. As far as the writer was informed, the conclusions were about as Dr. Chittenden has outlined in his paper published in this number. The association, it is understood, made it very clear to the dental colleges that they must come up to a certain standard and, failing in this, would not be regarded as acceptable institutions. Up to the close of the meeting some twenty-two colleges had accepted the conditions. If there be no further additions to this list, it will leave twenty-nine colleges in a peculiar position.

The Association of Faculties met in annual meeting, with forty-five members present.

It is difficult to determine the position that this body occupies at the present time. It was very evident that the members felt they had lost strength through the irregular action at St. Louis and were incapable of holding the membership to the rules, as had been the case for the past twenty-one years. The writer introduced a resolution at the opening meeting, with the object of repudiating the action of the meeting held at St. Louis. This was, as anticipated, laid on the table, but one member voting in the negative. This settled the question whether the Faculties intended to abide by its own constitution, violated at the aforesaid meeting. As they decided to confirm that action, they practically destroyed all the rules passed since 1884 for the government of the colleges under its jurisdiction. In doing this they eliminated all power once possessed over the educational institutions in membership.

That this was the feeling was very evident, for the several sessions lacked force. No business of any importance was transacted. The matter of fees claimed attention, and a motion was finally passed to raise the yearly fee to $150, beginning with the session 1906. It will probably be carried out by a few, but concert of action upon this minor matter is not to be expected.

A matter which would have been of vital importance to dental colleges under former conditions was a report from the Committee on Schools, Dr. A. O. Hunt, of Omaha, chairman. This was a very elaborate paper and closed with the following suggestions, "To secure a standard and have it properly administered, so that it will command the support of all interested.

"To select from this body ten persons, whose tenure of office shall be ten years (or, what is better still, for life), to be known as Dental Regents, with absolute authority to create and administer a standard for entering upon dental courses and requirements for graduation.

"That all schools of this association shall be bound by their action," etc.

Under the peculiar ruling of the acting president, a motion to adopt was permitted and carried, with the understanding that it be held over for next year for final action. As each session of the Faculties is a new body, it will puzzle the astute parliamentarians, at that time, to find a way to reconsider a question already decided at a previous annual meeting. It is not, however, probable that this very crude suggestion will pass then, or, if it should, that it can ever be enforced. There is not a dental school of any standing that would submit to any such interference with educational methods.

It is impossible to feel otherwise than discouraged at the future outlook for this body. It has performed a great work for the regeneration of dental education in this country during less than a quarter of a century of existence. The four men who called it into being in 1884 had no conception of the extended influence that this body would exert in the future upon dental education in the United States, moulding it into form and giving it an ethical character previously unknown. The life of all organizations is necessarily limited. The conflict of opinion at no very remote period weakens and causes disruption, and it is, therefore, not to have been expected that this body would have been an exception to the universal rule. It is, nevertheless, a cause of profound regret that it should thus early have entered a period of senile decay. Its power has passed into other hands, and, while it may linger for a time as a moribund body, its moral force in dental educational circles has departed, and those who have the training of young men must seek a broader ethical life for their standard of work.

The hospitality of the dental profession of Buffalo was not the least of the pleasant things that goodly city offered to visitors. Everything was done to make the sojourners feel at home, with the result that there was a unanimous feeling that Buffalo was an ideal convention city, and the dentists within its gates were worthy representatives of the true professional spirit.

